# Effects of dietary supplementation with polyphenols on meat quality in Saanen goat kids

**DOI:** 10.1186/s12917-018-1513-1

**Published:** 2018-06-11

**Authors:** Roberta Cimmino, Carmela M. A. Barone, Salvatore Claps, Ettore Varricchio, Domenico Rufrano, Mariangela Caroprese, Marzia Albenzio, Pasquale De Palo, Giuseppe Campanile, Gianluca Neglia

**Affiliations:** 1 Italian Buffalo Breeders Association, V. Petrarca 42/44, 81100 Caserta, Italy; 2 Department of AgriculturalSciences, Federico II University, Via Università 133, 80055 Portici, Naples Italy; 3 Research Centre for Animal Production and Aquaculture (CREA, S.S. 7 Appia, 85051, Bella Muro, PZ Italy; 4 Department of Sciences and Technologies, University of Sannio, V. Port’Arsa 11, 82100 Benevento, Italy; 5 Department of Agricultural Food and Environmental Sciences, University of Foggia, Via Napoli 25, 71122 Foggia, Italy; 6 Department of Veterinary Medicine, University “Aldo Moro” of Bari, S.P. per Casamassima, km 3, Valenzano, 70010 Bari, Italy; 7 Department of Veterinary Medicine and Animal Production, Federico II University, V. F. Delpino 1, 80137 Naples, Italy

**Keywords:** Polyphenols, Kids, Fatty acids, Sensory evaluation, Malondialdehyde

## Abstract

**Background:**

Diet supplementation with polyphenols is a novel strategy to improve meat quality in livestock, by preventing oxidative deterioration of lipids and protein. Polyphenols have beneficial effects on both human and animal health and can be obtained from several sources, such as olive mill wastewaters (OMWW). These are severe environmental pollutants and therefore may be recycled and utilized in other sectors. The aim of this study was to evaluate growth performance, meat characteristics, fatty acid composition, antioxidant status, different forms of myoglobin and malondialdehyde formation in kids who received a diet supplemented with polyphenols obtained from OMWW. Weaned goat kids (*n* = 18) were divided into two homogenous groups: control (C) group (*n* = 9) received a fattening standard diet while the other group (*n* = 9) received the same diet, supplemented with 3.2 mg/day of polyphenols powder extract (PE group). Average daily gain (ADG) was calculated 10 days apart throughout the study. After 78 days, the kids were slaughtered and pH and carcass yield were evaluated. *Longissimus thoracis et lumborum* muscle was collected and utilized for chemical analysis, meat quality evaluation and oxidative stability.

**Results:**

No differences were recorded in ADG, carcass weight, pH and dressing between the two groups. Furthermore a similar meat proximate composition, texture and color was observed. Dietary polyphenols supplementation significantly (*P* < 0.01) decreased short chains (<C12:0) (2.93 + 0.50 and 0.35 + 0.40 g/100 g of fatty acids, for C and PE Group, respectively), and saturated (49.22 ± 2.39 and 39.51 ± 1.95 g/100 g, in C and PE Group, respectively) fatty acids. Furthermore, a higher (*P* < 0.05) proportion of monounsaturated (34.35 ± 2.84 and 42.22 ± 2.32 g/100 g, in C and PE Group, respectively) fatty acids was recorded. Malondialdehyde formation was significantly (*P* < 0.05) lower in PE compared to C Group (0.25 ± 0.005 and 0.15 ± 0.005, in C and PE Group, respectively).

**Conclusions:**

Polyphenols dietary supplementation has positive effects on kid meat, improving fatty acid profile and reducing malondialdehyde contents. Furthermore the utilization of OMWW as the source of polyphenols may represent an innovative strategy to re-utilize agri-food industry wastes.

## Background

An increased awareness of the consumers was recently observed regarding red meat’s production and consumption. A typical example was the latest report of the World Health Organization (WHO), which classified the consumption of red meat as “*probably carcinogenic for man*” [1], although, as outlined by other authors [2], there are still a number of gaps in the current knowledge about this topic. Furthermore, eating red meat has been correlated with higher incidence of chronic diseases [3]. For this reason there has been an increased interest for new breeding techniques which confer potential health benefits to the consumers [4].

The supplementation of ruminants diet with antioxidants is considered an effective strategy for changing and ameliorating the fatty acid composition of meat in response to consumer demands [4]. In fact, it has been observed that the inclusion of linseed and fish oil on the lamb diet may have a negative impact on the oxidative stability and on physicochemical properties of the meat [5]. In addition, the changes that occur in the muscle during the post-mortem period may create an unbalanced proportion between the antioxidant and pro-oxidant capability, increasing the risks of oxidative damage [6]. The antioxidant status of the muscle is the main factor affecting oxidative deterioration in meat [7]. Oxidative deterioration of lipids and proteins in meat could adversely affect its nutritional quality and shelf life, reducing flavor, color and quality of meat, with a negative impact on meat consumption.

Thus, it is essential to preserve the quality and the safety of the meat by attenuating oxidative deterioration. Recently, the interest of food processing industries in the use of natural antioxidants rather than synthetic counterparts was increased, for either low environmental impact and economical reasons [8]. Furthermore natural antioxidants are well accepted by the consumers, because they are considered safe and healthy [9]. For these reasons, many studies have been carried out to develop new natural antioxidants, especially from plants [8], among which polyphenols. Polyphenols are a large family of more than 8000 natural compounds derived from plants and characterized by the presence of a phenol ring in their structure [10, 11]. Epidemiological, clinical and nutritional studies strongly support the evidence that dietary phenolic compounds are effective in the prevention of common diseases, including cancer, neurodegenerative diseases and gastrointestinal disorders [12]. Furthermore, a large number of studies suggest several immunomodulatory and anti-inflammatory properties of these compounds in humans [13, 14]. Some studies, performed in livestock, reported the positive effects of diet supplementation with polyphenols on pigs and chicken health. [11, 15, 16]. In addition, it has been hypothesized that dietary supplementation with polyphenols would increase beneficial lipids and oxidative stability of myoglobin and reduce the content of malondialdehyde (MDA) [see 11 for review].

Polyphenols can be obtained from several sources. One of these is the olive oil sector, particularly important in Mediterranean countries, such as Spain (the leading producer), Italy, Greece, Turkey, Syria and Tunisia. Olive mill wastewaters (OMWW) are the main pollutant by-phase and traditional suction systems Mills. The management of OMWW is a serious environmental issue for the presence of organic compounds that turn OMWW into phytotoxic materials [17]. Nevertheless, OMWW contains valuable resources, such as polyphenols, which can represent about 10% of OMWW dry weight [18]. Some olive oil derived-compounds, such as hydroxytyrosol, have an important role in preventing cardiovascular diseases [19]. Thus OMWW could be recycled and utilized in other sectors, such as animal feeding, to reduce its environmental impact.

Therefore, the objective of this study was to investigate the effects of the supplementation with polyphenols obtained from OMWW in the diet of Saanen goat kids on growth performance and meat quality. In particular, meat proximate composition, texture and colorimetric properties, fatty acid composition, lipid oxidation and the level of the different forms of myoglobin of *Longissimus thoracis et lumborum* (LTL) muscle were evaluated.

## Methods

### Animals, experimental design and diet composition

All experimental procedures were approved by the Ethical Committee on Animal Research of the University of Naples (Protocol number: 2014/0105988 of 1st December 2014), and the study was carried out in accordance with EU Directive 2010/63/EU for animal experiments.

The trial was performed on 18 Saanen female kids maintained in the experimental farm of CREA (Research Centre for Animal Production and Aquaculture) located in Bella (PZ), in the South of Italy, at 40°75′ latitude and 15°67′ W longitude, and 802 m above sea level. The kids were allowed natural suckling until weaning, that occurred at 69 days of age. After weaning the animals were divided into 2 homogeneous groups, according to age and live body weight (LW), recorded at both birth and weaning: Control (C) group (*n* = 9): received a fattening standard diet (Table 1); Polyphenols extract (PE) group (*n* = 9): received the same diet of control group, supplemented with a powder of polyphenols extract from OMWW (3.2 mg/day, see below). The amount of polyphenols powder was defined according to antioxidant activity (see below) and some studies performed in growing lambs [5].

**Table 1:** Ingredients (%) and proximate composition (% of dry matter) of the basal diet administered to the kids throughout the experimental period

Ingredients (%) of concentrate administered		
Maize meal	60	
Faba bean (*Viccia faba minor*)	15	
Alfalfa pellets	5	
Soybean meal	5	
Wheat bran	10	
Oat	5	
	Concentrate	Dehydrated alfalfa
Dry Matter (g/100 g weight)	87.5	85.6
Ash	2.7	8.3
Crude protein	11.8	19.5
Crude fiber	6.19	25.6
Neutral detergent fiber	18.2	37.5
Acid detergent fiber	8.9	34.3
Lignin	2.1	9.8
Ether extract	3.8	1.9
Non structural carbohydrates	63.5	25.6
Starch	56.8	–

The kids of each group were individually penned in boxes (1 m^2^) throughout the study that lasted 78 days. All kids had free access to water and received the same ration, consisting of alfalfa hay ad libitum and a starter concentrate administered in increasing amount according to the growth following the recommendation of National Research Council [20]. Feed was administered twice a day, at 8.00 and 16.00 and polyphenols powder was mixed on daily bases with the concentrate in the PE Group. The inclusion level of polyphenols extract was variable throughout the study according the amount of administered concentrate from 1.70% at the start of the trial to 0.83% in the last week (1.16% on average). Feed intake was determined from orts (refusals) collected daily in the morning (when present) before the next feed administration. The amount and the composition of orts were utilized to calculate the dry matter (DM) intake and the composition of the ingested diet. Individual feedstuff and orts were sampled every 15 days and the analyses were carried out as per AOAC (Association of Official Analytical Chemists) procedures [21] after drying at 65 °C and mechanical reduction of the samples (granulometry 1 mm for all analyses). The chemical composition of individual feedstuff is reported in Table 1.

Each animal was individually weighed 10 days apart and average daily gain (ADG) was calculated by dividing the difference between two consecutive LW measurements (LW1 and LW2) with the number of days elapsed (LW2 − LW1/days).

### Polyphenols determination

Polyphenols extract from OMWW was obtained and characterized as reported by Parrillo et al. [22]. Briefly, the polyphenolic extract was obtained by membrane separation of OMWW according to previous studies [23] and the main phenolic compounds were identified by HPLC (LC-4000 Series Integrated HPLC Systems, JASCO, Japan) according to Azaizeh et al. [18] (Table 2). Total phenols content in OMWW extract was determined by the Folin-Ciocalteu method [24]. The antioxidant activity of the OMWW extract was evaluated by using the free radical ABTS (2,2-Azino-bis-3-ethylbenzothiazoline-6-sulfonic acid), according to the procedures described by Re et al. [25].

**Table 2:** Main phenolic compounds of olive mill wastewaters (OMWW) extracts

Main compounds	mg/kg	Percent
Hydroxytyrosol	20,829	21.27
Flavonoid	3278	3.35
Tyrosol	3947	4.03
Caffeic acid	9991	10.20
Verbascoside	17,449	17.82
Hydroxytyrosol derivatives (OHTY-glycol, OHTY-glucoside, 3,4-diidrossifenilethanol – 3,4-DHPEA Elenolic acid mono-Aldehyde, 3,4-DHPEA– AC hydroxytyrosol acetate)	26,826	27.40
Verbascoside derivatives (isoverbascoside, β-hydroxyverbascoside,β-hydroxyisoverbascoside)	6498	6.64
Other derivatives of cinnamic acid (cinnamic acid, *o*-, *p*- coumaric acid, ferulic acid)	3372	3.44
Caffeic acid derivatives (chlorogenic acid, neochlorogenic acid, 1-*O-*caffeoylquinic acid, 3,5-*O-*dicaffeoylquinic acid)	4681	4.78
Other polyphenols	1055	1.08
Total polyphenols (TPC) (mg/kg)	97,926	
Antioxidant activity (mmolitrolox/kg sample)	8521	

### Slaughtering procedure and muscle sampling

At the end of the experimental period (147 days of age), all the kids were left overnight with ad libitum access to water and slaughtering procedures were carried out in accordance to the EU Regulation 2009/1099/EC on the protection of animals at the time of killing. The animals were stunned by captive bolt and the exsanguination from the jugular vein was carried out. After slaughtering, evisceration and dressing, each carcass was weighed and the pH was measured 45 min post-mortem in the LTL muscle (between 11th and 13th thoracic vertebra), using an automatic digital pH-metertest-205 (TestoInc, Sparta, NJ, USA), equipped with a penetrating electrode. The probe was calibrated with pH 4 and 7 standard buffer solutions. The dressed carcass comprises the body after removing skin, head, fore feet (at the carpal–metacarpal joint), hind feet (at the tarsal–metatarsal joint), lung, heart, liver, spleen, kidneys, kidney fat, and gastro-intestinal tract fat. Furthermore, the stomachs (rumen, reticulum, omasum, and abomasum) and the postruminal tract (intestine and caecum) were removed. Dressing percentage (DP) was calculated according to the following formula:

$$ \mathrm{DP}=\left(\mathrm{hot}\ \mathrm{carcass}\ \mathrm{weight}/\mathrm{live}\ \mathrm{weight}\right)\ \mathrm{x}\ 100 $$

All carcasses were stored at 2 °C for 24 h. Ultimate pH was assessed 24 h *post-mortem* on *LTL* muscle and the carcasses were weighed again. After that, a professional butcher removed the *LTL* muscle, between the sixth thoracic and 5th lumbar vertebra, from the right side of each carcass. *LTL* samples were stored at 4 °C and used for chemical analysis, meat quality evaluation and oxidative stability. Furthermore, pH was also assessed 48 h post-mortem on LTL muscle as described above.

### Chemical analyses

All analyses were carried out in the Laboratories of the Department of Veterinary Medicine and Animal Production (DMVPA), and Department of Agriculture Sciences (DIA) of University of Naples Federico II (Italy). The chemical composition of *LTL* muscle was determined on refrigerated (4 °C) samples according to the AOAC procedures [26] by using a Foodscan equipment (Food Scan™Lab 78,810). Each sample was analyzed in duplicate.

### Texture measurements

Tenderness was evaluated on meat cooked in a thermostatically controlled water bath at 90 °C on day 1 after slaughtering. To monitor temperature achieved in the middle of the sample (70 °C), a portable digital thermometer (TEMP7 thermometer digital microprocessor for Pt100 probes, TECNAFOOD MO, IT) was used. After cooking the samples were dried with paper, in order to eliminate the moisture on the surface, and held in a cooler at 4 °C before coring. A minimum of four cores of 1.27 cm^2^ from each LTL muscle were obtained parallel to the longitudinal orientation of the muscle fibers. The *Instron 5565* with a *Warner–Bratzler shear* (WBS) device and crosshead speed set at 100 mm/min and a load cell of 500 kg [27] was used. According to Girard et al. [28], the measured parameters were *Shear myofibrillar force* (SMF) and *Warner-Bratzler Shear Force* (WBSF) both expressed in kg*.* Indeed in a shear force curve some peaks of less importance may be observed, before and after maximum positive peak shear force (WBSF). Bouton & Harris [29] related these first small peaks to the myofibrillar component of shear force coinciding with initial yield (SMF). WBSF minus SMF estimates the connective tissue contribution.

### Instrumental color

The color of the meat was determined on the surface of samples using a U3000 spectrophotometer, equipped with integrating sphere (Hitachi, Tokyo, Japan). Although illuminant D_65_ is largely utilized, the use of Illuminant A is recommended by AMSA [30]. For this reason color coordinates, employing the CIEL*a*b* system with two illuminants, D_65_ (6500 K) and A (2856 K) and 10° standard observer, were Lightness (L*); redness (a*), and yellowness (b*) were also calculated, according to AMSA [30]. The color analyses were carried out in duplicate every day, from 24 h post-mortem until day 7, on samples (diameter 2.54 cm, thick 2 cm) stored at 4 °C. The samples were placed on white trays and wrapped with oxygen-permeable film.

Color difference (∆E*) between each day of storage and the day 0 was calculated as follows:

$$ {\Delta  \mathrm{E}}^{\ast }={\left({\Delta  \mathrm{L}}^{\ast 2}+{\Delta  \mathrm{a}}^{\ast 2}+{\Delta  \mathrm{b}}^{\ast 2}\right)}^{1/2} $$

Where AL*, ∆a^*^and ∆b^*^are the differences between L*, a* and b* values at time 0 and the individual readings each day.

Metmyoglobin (MMb), deoxymyoglobin (DMb) and oxymyoglobin (OMb) percentages were estimated according to [30] on the basis of the *Reflex Attenuance* (A) at the isobestic points 572, 525, 473 and 730 nm (nm). The *Reflex Attenuance* (A) was identified as:

$$ \mathrm{A}=\log\ \left(1/\mathrm{R}\right) $$

where R expresses the reflectance at a specific wavelength in decimal (0.30 rather than 30%).

Therefore from the *Reflex Attenuance* (A), it was possible to estimate the three forms of myoglobin:

$$ \%\mathrm{MMb}=\left[1.395-\left(\mathrm{A}572-\mathrm{A}730/\mathrm{A}525-\mathrm{A}730\right)\right]\ \mathrm{x}100 $$

$$ \%\mathrm{DMb}=\left[2.37{5}^{\ast}\right(1-\left(\left(\mathrm{A}473-\mathrm{A}730\right)/\left(\mathrm{A}525-\mathrm{A}730\right)\right]\mathrm{x}100 $$

$$ \%\mathrm{OMb}=\left[\right(100-\left(\%\mathrm{MMb}+\%\mathrm{DMb}\right)\Big] $$

### Fatty acid analysis

Fatty acid composition of the meat was assessed on fresh samples on day 1 post-slaughtering. The muscle was blended in a food processor and the lipids were extracted from 5 g samples in duplicate, using chloroform:methanol (2:1, *v*/v) [31]. The extracted lipids were transmethylated to their fatty acid methyl esters (FAME) according to Christie [32]. The amount of fatty acid (g/100 g of FAME) was determined by gas chromatography using a chromatograph (DANI fast GC, Italy), with a flame ionization detector and equipped with a capillary column (TR-CN 100) (60 m × 0.25 mm diameter × 20 μm). Helium was used as carrier gas at a flow rate of 1.2 mL/min. The split ratio was 50:1, the injector was set at 280 °C and the detector at 240 °C. The oven temperature was programmed and held at 80 °C for 5 min, then increased to 165 °C at 5 °C/min, held at 165 °C for 1 min, and then increased to 260 °C at 3 °C/min, and then held at 260 °C for 1 min.

Identification of each fatty acid was obtained by comparing the chromatogram with the reference standard mixture of Supelco 37 component series FAME MIX (Supelco Bellefonte, PA, USA) and a mixture of CLA isomers (Nu-Chek-Prep, Inc., Elysian, MN, USA). Retention time and area of each peak were calculated using the Clarity software (Clarity v.2.4.1.77, Data Apex Ltd., 2005). The atherogenic index (AI) and the thrombogenic index (TI) were obtained by using the following equations [33].

$$ \mathrm{AI}:\left[\left(4\ \mathrm{x}\ \mathrm{C}14:0\right)+\mathrm{C}16:0+\mathrm{C}18:0\right]/\left[\Sigma \mathrm{MUFA}+\Sigma \mathrm{PUFA}-\mathrm{n}6+\Sigma \mathrm{PUFA}-\mathrm{n}3\right] $$

$$ \mathrm{TI}:\left[\left(\mathrm{C}14:0+\mathrm{C}16:0+\mathrm{C}18:0\right)/\left(0.5\mathrm{xMUFA}\right)+\left(0.5\times \mathrm{PUFA}-\mathrm{n}6\right)+\left(3\mathrm{xPUFA}-\mathrm{n}3\right)+\left(\mathrm{PUFA}-\mathrm{n}3/\mathrm{PUFA}-\mathrm{n}6\right)\right] $$

### Evaluation of lipid oxidation

The evaluation of lipid oxidation in meat was based on the determination of malondialdehyde (MDA), a secondary lipid oxidation product (nmol/μg of meat). MDA was determined by a specific kit (Lipid Peroxidation (MDA) Assay Kit, Sigma-Aldrich, USA) according to the manual instructions. MDA was quantified by spectrophotometric analysis (Model 680 Microplate Reader, Biorad, Italy), at a wave length of 532 nm. MDA content was evaluated at 1, 3 and 7 days *post-slaughtering*.

### Statistical analysis

The experimental data were subjected to one way analysis of variance (ANOVA) using the linear model of the SAS software [34], following confirmation of normality and homogeneity of variance. The influence of dietary treatment (polyphenols supplemented diet or control diet),storage (day 1, 3 and 7) and their interaction (dietary treatment x storage) were used as main factors to analyze color parameters, MDA levels and the different forms of myoglobin. Results are presented as mean values with standard error of mean (SEM). The mean were compared using the Student’s *t*-test. The differences were considered significant when *P* values were lower than 0.05, whereas a tendency was considered for *P* < 0.10.

## Results

### Animal performance and carcass characteristics

The effects of polyphenols supplementation on growth performance and carcass properties are shown in Table 3. A similar ADG from weaning to slaughter and a similar weight at slaughter were observed in the two Groups. Furthermore, carcass weights and yields were similar between groups at 0 and 24 h post mortem (Table 3). No significant differences were observed in the pH decline values between the groups both at 45′ minutes and 24 h post-mortem (Table 3).

**Table 3:** Age and weight at weaning and at slaughter, average daily gain (ADG), feed consumption, carcass characteristics and dressing percentage in Control (C) and polyphenols extract (PE) group (mean values ± SEM)

	Groups		Significance
	C	PE	P
Age at weaning (days)	69.8 ± 1.30	68.9 ± 1.50	0.65
Age at slaughtering (days)	146.8 ± 1.30	145.9 ± 1.50	0.65
Weaning body weight (kg)	11.0 ± 0.75	10.7 ± 0.86	0.78
Final body weight (kg)	17.9 ± 0.99	18.6 ± 1.28	0.69
Weight gain (kg)	6.94 ± 0.60	7.93 ± 0.61	0.26
ADG (kg/g)	0.09 ± 0.01	0.10 ± 0.01	0.26
Concentrate intake (kg/day)	0.30 ± 0.00	0.29 ± 0.00	0.33
Hay intake (kg/day)	0.68 ± 0.01	0.67 ± 0.01	0.39
DM intake (kg/day)	0.84 ± 0.02	0.85 ± 0.03	0.57
Carcass weight at slaughtering (kg)	10.0 ± 0.51	10.0 ± 0.77	0.98
Carcass weight at 24 h (kg)	9.7 ± 0.50	9.7 ± 0.74	0.99
pH at slaughtering	6.8 ± 0.11	6.8 ± 0.12	0.90
pH at 24 h	5.8 ± 0.03	5.8 ± 0.03	0.64
Dressing at slaughtering (%)	55.8 ± 1.41	54.0 ± 0.48	0.15
Dressing at 24 h (%)	54.6 ± 1.39	52.3 ± 0.45	0.14

### Meat quality

A similar meat chemical composition was recorded in C and PE group (Table 4).

**Table 4:** Proximate meat composition of LTL muscle in control (C) and polyphenols extract (PE) group (mean values ± SEM)

	Groups		Significance
	C	PE	P
Moisture (%)	66.6 ± 0.46	64.1 ± 0.35	0.11
Fat (%)	13.7 ± 0.46	16.5 ± 0.31	0.09
Protein (%)	18.3 ± 0.30	18.7 ± 0.40	0.43
Collagen (%)	2.3 ± 0.28	3.0 ± 0.16	0.14
Ash (%)	0.91 ± 0.12	0.84 ± 0.05	0.58
pH*	6.1 ± 0.01	6.2 ± 0.01	0.51

*pH was assessed on LTL sample 48 h after slaughtering

The texture assay highlights a double peak (SMF = 1st peak and WBSF = 2nd peak)in 9/9 animals of C samples compared to 3/9 animals of PE counterparts. SMF values obtained by deformation curves where a double peak was evident, tended to be higher (*P* = 0.06) in PE compared to C group, whereas similar WBSF values were recorded (Table 5).

**Table 5:** *Shear myofibrillar force* (SMF) and *Warner-Bratzler Shear Force* (WBSF) recorded in the LTL muscle of control (C) and polyphenols extract (PE) group (mean values ± SEM)

	Groups		Significance
	C	PE	P
WBSF, kg	4.5 ± 0.10	4.6 ± 0.10	0.86
SMF, kg	0.53 ± 0.07	1.14 ± 0.33	0.06

**Fig. 1:**
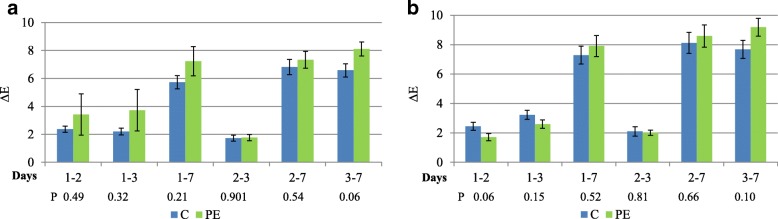
Difference of color (∆E) by 1 to 7 days of storage with illuminant D65 (**a**) and illuminant A (**b**), in control (**c**) and polyphenols extract (PE) group (blue) and treated (green) group. The error bars represent standard error

The color coordinates detected by using the two illuminants (A and D65) were similar between the two groups. In fact, although the illuminant A would give more emphasis on the proportion of red wave lengths, no significant differences were recorded in lightness (L*); redness (a*), yellowness (b*) between the two groups and throughout the storage. Furthermore, no significant interaction storage x treatment was assessed. Slight differences were observed in color differences (∆E*) during the first three days of storage in the meat of PE Group compared to the control when analyzed by illuminant A, while an opposite trend was recorded with the illuminant D65. In any case, no statistical differences were recorded (Fig. 1) and subsequently the changes showed the same trend with both illuminants.

Neither dietary treatment (*P* = 0.77 and *P* = 0.99, for OMb and MMb, respectively) nor storage period (*P* = 0.33 and *P* = 0.10, for OMb and MMb, respectively) and their interaction (*P* = 0.98 and *P* = 0.95, for OMb and MMb, respectively) influenced the estimated levels of myoglobin forms. As expected, during the storage period, the estimated level of MMb tended to increase in both groups, while the levels of OMb decreased until 3 days and increased after 7 days of storage (Table 6).

**Table 6:** Estimated levels (mean percentage ± SEM) of metmyoglobin (MMb) and oxymyoglobin (OMb), in the LTL muscle of control (C) and treated (PE) group, throughout the storage at 4 °C

	Groups	1	2	3	7
MMb	C	40.73 ± 1.88	45.28 ± 1.18	53.57 ± 1.67	61.78 ± 3.60
	PE	40.99 ± 1.94	47.67 ± 1.52	54.41 ± 1.88	63.37 ± 3.94
OMb	C	68.93 ± 2.70	34.04 ± 3.04	15.07 ± 3.24	43.87 ± 9.42
	PE	62.66 ± 7.02	38.13 ± 4.83	21.42 ± 5.69	51.48 ± 10.28

### Fatty acid composition and oxidative stability

The fatty acids composition of LTL intramuscular lipids in the two groups is shown in Table 7. Dietary polyphenols supplementation determined a decrease of short chain fatty acids (<C12:0) as well as of Myristic (C14:0) Myristoleic (C14:1), Pentadecanoic (C15:0), Palmitic (C16:0) and Palmitoleic (C16:1) acids.

**Table 7:** Mean fatty acid composition (g/100 g of FAME) of LTL muscle in control (C) and polyphenols extract (PE) group (mean values ± SEM)

	Groups		Significance
Fatty Acids	C	PE	P
< C12:0	2.93 ± 0.50 ^B^	0.35 ± 0.40 ^A^	0.001
C12:0	0.45 ± 0.36	0.20 ± 0.07	0.06
C14:0	4.00 ± 0.58 ^b^	2.28 ± 0.47 ^a^	0.04
C14:1	0.46 ± 0.06 ^B^	0.23 ± 0.05 ^A^	0.01
C15:0	0.58 ± 0.06 ^b^	0.41 ± 0.05 ^a^	0.04
C16:0	23.81 ± 0.85^B^	20.22 ± 0.70^A^	0.01
C16:1	3.05 ± 0.41 ^b^	1.63 ± 0.33 ^a^	0.02
C17:0	1.12 ± 0.17	1.17 ± 0.14	0.79
C17:1	1.33 ± 0.17	0.91 ± 0.14	0.08
C18:0	15.60 ± 1.25	14.31 ± 1.02	0.43
C18:1 cis	27.16 ± 3.15 ^a^	36.01 ± 2.57 ^b^	0.05
C18:1 trans	0.08 ± 0.73	1.64 ± 0.60	0.12
C18:2 cis	12.22 ± 1.07	13.64 ± 0.88	0.32
C18:2 trans n6	0.48 ± 0.05	0.37 ± 0.04	0.09
C18:3 n6 γ-linolenic	0.10 ± 0.03	0.07 ± 0.02	0.43
C20:0	0.05 ± 0.04	0.03 ± 0.03	0.74
C20:1	1.62 ± 0.15	1.59 ± 0.12	0.89
C20:2 n6	0.10 ± 0.03	0.05 ± 0.02	0.31
CLA cis 9–trans 11	1.22 ± 0.12	1.02 ± 0.10	0.24
C20:3 n3	1.00 ± 0.40	1.22 ± 0.33	0.67
C20:4 n6	0.72 ± 0.75	1.63 ± 0.61	0.36
C22:0	0.32 ± 0.12	0.27 ± 0.10	0.76
C22:6 n3	0.63 ± 0.40	0.24 ± 0.33	0.47
C24:0	0.33 ± 0.20	0.26 ± 0.16	0.77
C24:1	0.63 ± 0.38	0.20 ± 0.31	0.39
PUFA n6	13.62 ± 0.96	15.77 ± 1.30	0.31
PUFA n3	1.63 ± 0.75	1.47 ± 0.61	0.86
n6/n3	8.36 ± 0.86	10.73 ± 0.91	0.41
ΣSFA	49.22 ± 2.39^B^	39.51 ± 1.95^A^	0.01
ΣMUFA	34.35 ± 2.84^a^	42.22 ± 2.32^b^	0.05
ΣPUFA	16.47 ± 2.02	18.26 ± 1.65	0.51
UFA:SFA	1.08 ± 0.13 ^A^	1.55 ± 0.07 ^B^	0.01
PUFA:SFA	0.34 ± 0.04	0.47 ± 0.05	0.09
AI	1.28 ± 0.19 ^b^	0.69 ± 0.16 ^a^	0.03
TI	2.13 ± 0.32^B^	1.38 ± 0.26 ^A^	0.01

Different superscript letters within the same row indicate significant differences (^a.,b.^: *P* < 0.05; ^A.,B.^: *P* < 0.01)

Note: Σ SFA = (<C12:0 + C12:0 + C14:0 + C15:0 + C16:0 + C17:0 + C18:0 + C20:0 + C22:0 + C24:0), Σ MUFA = (C14:1 + C16:1 + C17:1 + C18:1cis + C18:1trans + C20:1 + C24:1), Σ PUFA = (ΣCLA+Σn3 + Σn6),

UFA:SFA = [(ΣMUFA+ΣPUFA)/ΣSFA],

PUFA:SFA = (ΣPUFA/ΣSFA),

AI: [(4 x C14:0) + C16:0 + C18:0] / [ΣMUFA +ΣPUFA-n6 + ΣPUFA-n3],

TI: [(C14:0 + C16:0 + C18:0) / (0.5xMUFA) + (0.5xPUFA-n6) + (3xPUFA-n3) + (PUFA-n3 / PUFA-n6)]

A significant lower (*P* < 0.01) percentage of saturated (SFA) and higher (*P* < 0.05) proportion of monounsaturated (MUFA) fatty acids were recorded in PE group compared to C group. However, no differences were recorded in the total amount of polyunsaturated fatty acids (PUFA), while the unsaturated fatty acids (UFA):SFA ratio was influenced by the treatment (*P* < 0.01). Furthermore, the PUFA:SFA (P:S) ratio tended to be higher (*P* = 0.09) in PE group, compared to C group. Therefore, the meat of PE group was also characterized by a significant lower AI index (*P* < 0.05) and TI index (*P* < 0.01), compared to the control counterparts.

**Fig. 2:**
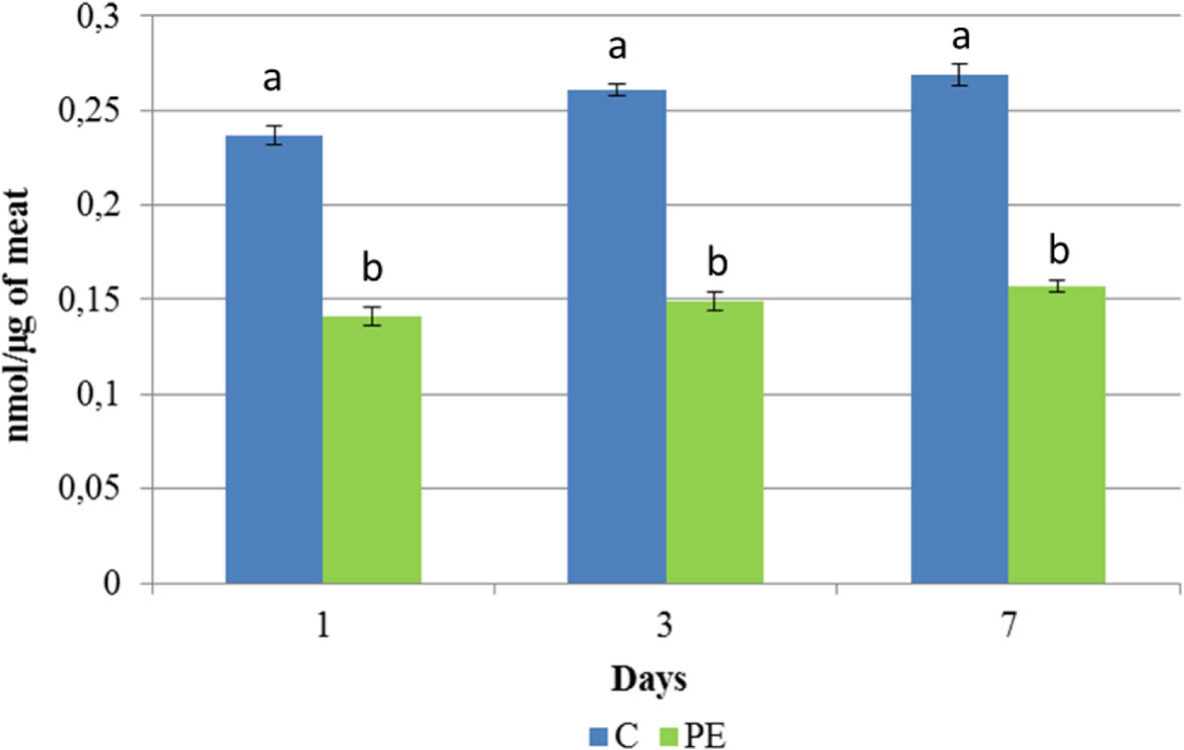
Lipid peroxidation in meat during the days of storage (expressed in MDA nmol/μg of meat), the error bars represent standard error

In relation to lipid oxidation, a significant effect of dietary treatment was observed. In particular, lower (*P* < 0.05) MDA values were recorded in PE group, compared to C group (0.25 ± 0.005 and 0.15 ± 0.005 in Group C and PE, respectively), as showed in Fig. 2, whereas neither the storage (*P* = 0.96) nor the interaction storage x dietary treatment (*P* = 0.97) influence the results.

## Discussion

This study aimed to ascertain the influence of dietary polyphenols administration on growth performance and meat quality in kids. Contrasting results have been reported on the effects of plant extracts administration on live weight gain, carcass weight and dressing percentage either in ruminants [35, 36], and not ruminants [11, 37]. According to some authors [37] no adverse effect on growth performance or protein and aminoacids digestibility were recorded in broilers after 2.5 g/kg of grape seed extracts administration, while a delayed growth rate was observed after 5 g/kg of supplementation. On the contrary, other authors [38] report an increase of final body weight and DWG in broilers fed dietary polyphenol-rich grape seed. Few studies have been performed in ruminants till now. Similar growth performance were observed in growing lambs fed a diet supplemented with grape pomace, vitamin E or grape seeds extract [39]. Similarly, the supplementation of the diet with pomegranate seed pulp [40] or with vitamin E, Turmeric powder or *Andrographis paniculata* powder [35] did not affect the growth of kids.

Also in our study, growth performance was not influenced by dietary polyphenols supplementation, but some aspects need to be considered. First of all about 8000 phenolic compounds have been identified in different plant species [11] and in different amounts, creating serious difficulties in making a comparison among the studies. Furthermore, fewer information is present on bioavailability of phenolic compounds in ruminants and their effects on bacterial rumen population, fermentation and absorption [10] compared to non-ruminants [11]. It can not be ruled out that different compounds and/or different amounts of polyphenols may lead to different results.

Also the proximate composition of the meat in our trial was not influenced by dietary polyphenols supplementation. This result is in agreement with previous studies carried out in goat, in which dietary herbal antioxidants [35] or vitamin E [36] were administered. Similarly, vitamin E supplementation did not affect carcass traits and dressing percentage in lambs [41]. Regarding the texture, the meat of control kids showed the occurrence in all samples of an initial yield putatively related to the myofibrillar component of shear force and classified as shear myofibrillar force [29]. On the contrary, a single peak was recorded in 6/9 animals of the PE group. For this reason, SMF values tended to be higher in PE group compared to C group. However, no differences were recorded for WBSF. This last result may indicate a similar collagen content of the meat recorded in the two groups. The evaluation of the contribution of either myofibrillar and connective tissue to meat texture after the cooking process can not be obtained without performing additional measurements [28]. Some authors [42, 43] suggested that the connective tissue contribution may be recorded by subtracting the initial yield from the shear force peak: although the shear force does not accurately represent the contribution of connective tissue to muscle tenderness [27, 44, 45], it may be hypothesized a larger contribution of the connective component to the final hardness in the control group, compared to the PE counterparts. To our knowledge, few studies have been performed to evaluate texture parameters in small ruminants after polyphenols diet supplementation. Carnosic acid supplementation seems to be an useful tool to improve meat sensory characteristics in fattening lambs, similarly to Vitamin E [46]. On the contrary, quercetin dietary supplementation seemed to worsen texture in fattening lambs [47].

No differences were observed in our study on color stability between the two groups, in accordance with previous reports carried out in lambs fed naringin supplementation [48]. The color is an important meat quality attribute because it is the first aspect which attracts consumers when choosing fresh meat. Color stability is mainly due to the increase in myoglobin oxidation and consequent metmyoglobin formation and accumulation. Kid’s meat is generally characterized by lower a* values compared to beef and lamb meat, outlining the difference in myoglobin concentration that reduces the discoloration. The effects of antioxidants on color stability in small ruminants have been analyzed in several studies in both kids [36, 40, 49] and lambs [39, 50] with contrasting results. According to some authors [40] no differences were recorded in kids fed pomegranate seed pulp on L*, while an increase of a* together with a decrease of b* was assessed. Similarly, no influence of vitamin E supplementation on color parameters was recorded in kids reared both in pen groups or maintained on pastures, although the rearing system significantly influenced meat color [36]. Dietary supplementation with antioxidants (vitamin E and flavonoids in particular) influences color parameters in some [39, 51], but not all [50] studies performed in growing lambs. Other authors reported a significantly lighter meat after polyphenols administration in lambs, probably because of their action in chelating iron and consequent low haemoglobin concentration [52]. Definitely, the results obtained in this study suggest that the concentration of MMb and OMb in meat, and consequently meat color, was not affected by dietary polyphenols supplementation. Only a slight lower, although not significant, ∆E* was observed in PE group, when detected by illuminant A. The latter determined higher value placing more emphasis on the proportion of red wave lengths. This aspect is important when the differences between treatments have to be highlighted, as outlined by AMSA [30]. Similar results were obtained by other authors [7] by using a dietary supplementation with a blend of 80% canola oil and 20% palm oil, and were justified by physical changes that occur during the storage, and not directly related to the oxidative process [49], since no significant effects on color and oxidative stability of myoglobin was recorded. However, it can not be ruled out that the amount of dietary polyphenols was not able to highlight significant effects, thus further studies are needed to evaluate different dosages.

In our study polyphenols extract supplementation affected the fatty acid profile of the meat. In particular, a significantly higher concentration of MUFA, together with a reduction of SFA was observed in PE Group compared to control, but no effects were recorded on PUFA. The acidic composition of meat in ruminants is generally different from that of non-ruminants. The PUFA/SFA ratio is lower because of the hydrogenation of UFA in the rumen, while this process does not occur in monogastrics that absorb UFA without any transformation in the gastrointestinal tract. Therefore, several UFA that are normally present in the diet of ruminants, are saturated and can not be present in derived food [53]. Thus, the diet plays the main role in the modification of fatty acid composition and rumen microbial population, as demonstrated by the evidence that rumen population activity on dietary UFA hydrogenation depends by the administered diet [54]. Several studies carried out in vitro [55–57] demonstrated that some phenolic compounds can modulate rumen fermentation, reducing the rate of biohydrogenation of fatty acids. In particular, some plants with high polyphenols content would be able to lower the biohydrogenation of PUFA in the rumen, increasing their by-pass and, consequently, the formation of CLA [56]. Similarly, an increase of C18 PUFA was recorded after 24 h in vitro incubation of an experimental diet with leaf fractions of papaya, extracted with hexane or chloroform [57]. However, only an increase of C18:1 (oleic acid) was recorded in our study, together with a similar n6 and n3 PUFA and a similar concentration of CLA, although a slight increase of C18:1 trans was observed. It is known that the presence of CLA in the tissues depends from the desaturation of C18:1 *trans-11*, catalyzed through Δ9 desaturase [58]. However, the amount of C18:1 *trans-11* in ruminant meat and milk is influenced by its hydrogenation to stearic acid (C18:0) or other C18:1 isomers at the rumen level by the microbial population [59]. It can not be ruled out that the amount of polyphenols extract utilized in our study might need to be increased to further improve the fatty acid profile of the meat. In a recent study [36], a lower amount of SFA, together with an increase of MUFA and lower AI and TI indexes were observed in kids after 450 mg/kg of vitamin E diet supplementation compared to control counterparts. Adeyemi et al. [4], reported a lower concentration of C16:0 and C16:1 n7 in goats fed 4 and 8% oil blend compared with control, justified by displacement or dilution effects of other fatty acids and by the decrease in the activity of lipogenic enzymes responsible for the synthesis of medium chain FA or the preferential incorporation of long chain FA from diet and/or adipose tissues. In a recent trial [5] dietary supplementation with red wine extract significantly increased the levels of C20:5 n3 in lambs, but no influence on oxidative stability was recorded. In any case, the P:S ratio, recognized as a healthy index, tended to be higher in PE group compared to control. Because of the hydrogenation of PUFA by the microbial population, the meat of ruminants is usually characterized by a low P:S ratio [60], leading to an increased risk of cardiovascular diseases. Therefore, the modification of fatty acid profile obtained in the present study suggests that dietary polyphenols administration may result in kid meat with beneficial effects on human health.

A further positive effect of polyphenols administration was recorded on oxidative stability of the meat, as demonstrated by the reduction of MDA, an aldehyde commonly utilized as marker of secondary lipid oxidation in meat and characterized by mutagenic and carcinogenic properties. In fact, MDA changes the link between lipoproteins and scavenger receptors on the surface of macrophages causing cholesterol ester buildup and foam cell formation and may also react with deoxyadenosine and deoxyguanosine, nitrogenous DNA bases, potentially causing mutations [61].

Although the values recorded in our study may be considered a limiting point from where rancid flavor overpowers flavor in beef [62] and MDA concentration is probably overestimated by spectrophotometric method [63], a significant decrease was observed in PE group compared to C group.

The use of antioxidant substances, such as high polyphenols content in the diet, reduces lipid peroxidation and the formation of DNA additives, by inhibiting the formation of MDA. A possible explanation of the antioxidant activity of polyphenols can be sought in the potential of chelating metal ions [64]: they are able to bind iron, forming insoluble compounds at the gastrointestinal lumen level and making it unavailable for absorption. Iron has been recognized as the most likely catalyst to promote lipid peroxidation by the formation of free radicals formed following the Fenton reaction [65]. This is supported by several studies carried out in chicken poultry [37], rats [66] and pigs [67], in which the inclusion in the diet of polyphenols obtained by different sources significantly reduces plasma iron concentration. Therefore, the reduction of iron absorption may explain the lower MDA content recorded in PE group compared to C group. However, it can not be ruled out that this phenomenon may be due to an enhancement of muscle oxidative stability, via an increased expression of Δ9 desaturase enzyme, as demonstrated in lambs fed tannins supplementation [68], or to the increase of endogenous antioxidant defense [69]. In any case, our results are in agreement with other studies in which a significant reduction in MDA formation following polyphenols administration was recorded in goat [40], chicken [70] and turkey meat [71].

## Conclusions

In conclusion, although no effects were observed on meat proximate composition, texture and colorimetric properties, the results of this study demonstrated that the supplementation of the diet with polyphenols extracts by OMWW is able to improve the fatty acid profile of kid meat, together with a reduction of MDA content. The utilization of this source of polyphenols may represent a novel strategy to re-utilize agri-food industry wastes as additives for livestock, meeting either meat industry and consumers requests of natural additives in animal farming. In any case, further studies are needed to test different doses of polyphenols and to better understand the role that the different categories of these compounds may have on meat quality.

## Abbreviations

∆E*: Color difference
A: Reflex attenuance
a*: Redness
ADG: Average daily gain
AI: Atherogenic index
ANOVA: analysis of variance
AOAC: Association of official analytical chemists
b*: Yellowness
C group: Control group
CREA: Research centre for animal production and aquaculture
DIA: Department of agriculture sciences
DM: Dry matter
DMb: Deoxymyoglobin
DMVPA: Department of veterinary medicine and animal production
DP: Dressing percentage
FAME: Fatty acid methyl esters
L*: Lightness
LTL: Longissimus thoracis et lumborum
LW: Live body weight
MDA: Malondialdehyde
MMb: Metmyoglobin
MUFA: Monounsaturated fatty acid
OMb: Oxymyoglobin
OMWW: Olive mill wastewaters
PE group: Polyphenols extract group
PUFA: Polyunsaturated fatty acid
SEM: Standard error of mean
SFA: Saturated fatty acid
SMF: Shear myofibrillar force
TI: Thrombogenic index
TPC: Total polyphenols
UFA: unsaturated fatty acid
WBS: Warner–bratzler shear
WBSF: Warner-bratzler Shear Force
